# Scene-Aware Correction of Scalar-to-Vector Reflectance in the Visible NO_2_ Retrieval Window

**DOI:** 10.3390/s26217084

**Published:** 2026-11-09

**Authors:** Yuqian Zhang, Kun Wu, Yijing Lu

**Affiliations:** 1 Collaborative Innovation Center on Forecast and Evaluation of Meteorological Disasters (CIC-FEMD)/State Key Laboratory of Climate System Prediction and Risk Management (CPRM)/Earth System Modeling Center, Nanjing University of Information Science and Technology, Nanjing 210044, China; 2 Department of Earth and Planetary Sciences, Institute for Atmospheric and Climate Science, ETH Zürich, 8092 Zürich, Switzerland; 3 Department of Earth and Atmospheric Sciences, Cornell University, Ithaca, NY 14853, USA

**Keywords:** polarization correction, vector radiative transfer, visible NO_2_ retrieval window, machine learning

## Abstract

**Highlights:**

**What are the main findings?**

- Scalar DISORT reflectances show systematic, scene-dependent differences from vector VLIDORT Stokes-I reflectances across wavelength, viewing geometry, surface albedo, NO_2_ column amount, and aerosol type; the uncorrected scalar baseline has an RRMSE of 3.0620%.
- The unified Transformer provides the most accurate correction, reducing the RRMSE by 92.15% to 0.2402%, while unified BP offers the lowest online inference time; correction-model selection should be application-dependent, while scene-specific models do not consistently outperform unified models.

**What are the implications of the main findings?**

- A paired scalar–vector correction can retain the efficiency of scalar radiative transfer while substantially reducing polarization-related forward-model errors in high-throughput visible NO_2_ retrievals.
- Polarization correction should be considered and quantified in the forward-model error budget of visible NO_2_ retrievals, and its residual error should be evaluated under withheld geometries and atmospheric scenes before being propagated to slant-column and vertical-column uncertainties.

**Abstract:**

Polarization affects forward modeling in the 420–455 nm visible NO_2_ retrieval window, yet operational retrievals require computational efficiency. We generated 205,200 paired scalar DISORT and vector VLIDORT simulations for a clear atmosphere and four pure-aerosol scenes (sulfate, sea salt, soot, and mineral dust), covering four NO_2_ vertical column densities, 19 wavelengths, viewing geometries, and surface albedos. Vector Stokes-I reflectance was the reference. We compared scene-specific inverse-distance-weighted look-up tables (IDW-LUTs) with unified and scene-specific backpropagation (BP), histogram-based gradient boosting (HGB), and Transformer models. Test samples were held out by geometry–surface state. The scalar baseline yielded an RRMSE of 3.0620%. IDW-LUT, unified BP, and unified HGB reduced this error by 44.90%, 79.48%, and 82.27%, respectively. The unified Transformer performed best (MARE = 0.1133%; RRMSE = 0.2402%; P95 = 0.3008%; 92.15% RRMSE reduction), at 0.4636 ms per 19-wavelength spectrum and 0.1159 MiB storage. The unified Transformer offers a strong accuracy–storage balance, while unified BP was the fastest among the unified machine-learning models. Conclusions are limited to clear-sky, pure, known-aerosol scenes; mixed aerosols, clouds, profile variability, instrument effects, and end-to-end NO_2_ retrievals remain to be tested.

## 1. Introduction

Nitrogen dioxide (NO_2_) is a short-lived, chemically reactive trace gas produced mainly by combustion and photochemical oxidation. It is both a harmful air pollutant and an important precursor of ozone and secondary aerosol. Its distribution therefore connects emission control, human exposure, atmospheric chemistry, and air-quality processes relevant to climate. Because NO_2_ varies strongly over urban and industrial source regions and on synoptic time scales, satellite observations complement sparse surface networks and provide quantitative constraints on emission inventories and trends. Differential optical absorption spectroscopy (DOAS) and related UV–visible methods use the structured NO_2_ absorption spectrum to retrieve slant columns and, with a radiative-transfer-based air-mass-factor calculation, tropospheric vertical columns [1,2,3,4].

The forward model is therefore as important to NO_2_ inversion as the spectral fitting algorithm. A typical retrieval represents the measured radiance or reflectance as the combined result of molecular absorption, Rayleigh scattering, aerosol scattering and absorption, surface reflection, viewing geometry, and instrumental response. A spectrally structured forward-model bias can be mistaken for NO_2_ absorption and then propagate into the slant column, air-mass factor, and vertical column. This matters for satellite products because small scene-dependent biases are repeated over millions of pixels and can appear as emission trends or differences between products [3,5].

The 420–455 nm window is useful for NO_2_ retrieval because it contains pronounced NO_2_ absorption and receives sufficient solar radiation for passive remote sensing. The signal is nevertheless controlled by more than absorption. Rayleigh scattering, aerosol extinction and phase-function effects, surface albedo, multiple scattering, wavelength-dependent cross sections, and the instrument line shape all contribute. Their influence changes with solar and viewing zenith angles, relative azimuth, surface brightness, aerosol loading and type, and the NO_2_ vertical column. Spectroscopy, atmospheric and aerosol states, spatial heterogeneity and radiative-transfer approximations can therefore interact in the fitted spectrum rather than contribute as independent errors [6,7,8,9].

Scalar and vector radiative-transfer models occupy different points on the accuracy–efficiency scale. A scalar solver such as DISORT propagates radiance intensity and is generally fast enough for repeated forward calculations and large retrieval archives [10]. Atmospheric scattering, however, produces a polarized Stokes field, and multiple scattering and surface reflection can feed that polarization back into the intensity. A vector solver such as VLIDORT retains this coupling and provides a more complete description of the top-of-atmosphere signal [11,12,13,14]. The additional Stokes components and polarization-dependent scattering operations increase the computational cost. Moreover, the scalar–vector difference is not a fixed offset: it changes with wavelength, geometry, surface albedo, aerosol optical properties, and NO_2_ column amount.

One practical compromise is to calculate the scalar solution online and apply a scalar-to-vector correction obtained from paired simulations. Look-up tables are transparent and easy to validate, and inverse-distance interpolation is inexpensive between sampled nodes. Their limitations become more evident as the number of controlling variables grows: resolving a high-dimensional state space increases storage, while sparse or strongly nonlinear regions can produce interpolation errors. A LUT also requires choices about node placement, normalization, neighborhood size, and extrapolation. Neural networks and other machine-learning (ML) surrogates provide a more compact alternative by representing nonlinear interactions without storing every grid node [15,16]; previous radiative-transfer emulators and scalar-approximation corrections demonstrate the potential of this approach [17,18,19,20].

Recent advances in machine learning have expanded the range of approaches available for structured tabular data. Gradient-boosted decision-tree methods, such as XGBoost and LightGBM, provide efficient approaches to nonlinear regression [21,22], while TabNet employs sequential attention-based feature selection to learn informative representations from tabular inputs [23]. Transformer-based architectures have also demonstrated the potential of attention mechanisms for modeling interactions among heterogeneous input features, although no single model family consistently outperforms all alternatives across tabular prediction tasks [24]. Beyond general tabular learning, deep neural networks have been applied to estimate atmospheric precipitable water vapor from meteorological reanalysis and observational data [25], while deep-learning-based methods have been developed to predict atmospheric turbulence in satellite-to-ground laser communication [26]. These studies illustrate the growing use of machine learning in atmospheric and optical modeling. In the present study, the scalar-vector reflectance difference depends on nonlinear interactions among wavelength, NO_2_ vertical column density, viewing geometry, surface albedo, scalar reflectance, and aerosol properties. This motivates the comparative evaluation of fully connected neural networks, tree-based ensembles, and attention-based architectures for efficient polarization correction.

ML correction also introduces its own risks. A model trained across heterogeneous aerosol scenes may average over scene-specific angular or spectral behavior, whereas separate models reduce the data available to each scene and add deployment and maintenance overhead. Tree ensembles and neural networks can be sensitive to feature scaling, an imbalanced training distribution, extrapolation beyond the simulated domain, and leakage between correlated spectra. Their predictions may be difficult to interpret physically, may lack calibrated uncertainty, and may become nonphysical for mixed or misclassified aerosol scenes absent from training. Reported accuracy also depends on whether the test set contains genuinely held-out geometry–surface states and whether prediction error is assessed together with latency and stored model size. These considerations motivate a direct comparison between a unified model that receives aerosol identity as an input and scene-specific models trained within each atmospheric regime.

Here we develop and evaluate scalar-to-vector corrections for the visible NO_2_ retrieval window. Paired scalar DISORT and vector VLIDORT simulations were generated for an aerosol-free atmosphere and four single-aerosol scenes (sulfate, sea salt, soot, and mineral dust) over 420–455 nm, multiple NO_2_ vertical columns, viewing geometries, and surface albedos. Vector Stokes-I reflectance is treated as the reference, and each method estimates the scalar-vector reflectance difference used as the polarization-correction target ΔR_pol_ = R_v_ − R_s_ before reconstructing the vector-equivalent reflectance. Five scene-specific inverse-distance-weighted LUTs are compared with unified and scene-specific BP, histogram-based gradient boosting, and Transformer models. We examine the size and structure of the scalar–vector discrepancy, compare unified and scene-specific ML on held-out geometry–surface states, and quantify the associated offline preparation time, online inference time, and stored artifact size. Section 2 describes the simulations, data partitioning, correction methods, and metrics; Section 3 presents the physical error structure and test results; Section 4 discusses accuracy–efficiency trade-offs and limitations; and Section 5 is the conclusion.

## 2. Materials and Methods

**Figure 1:**
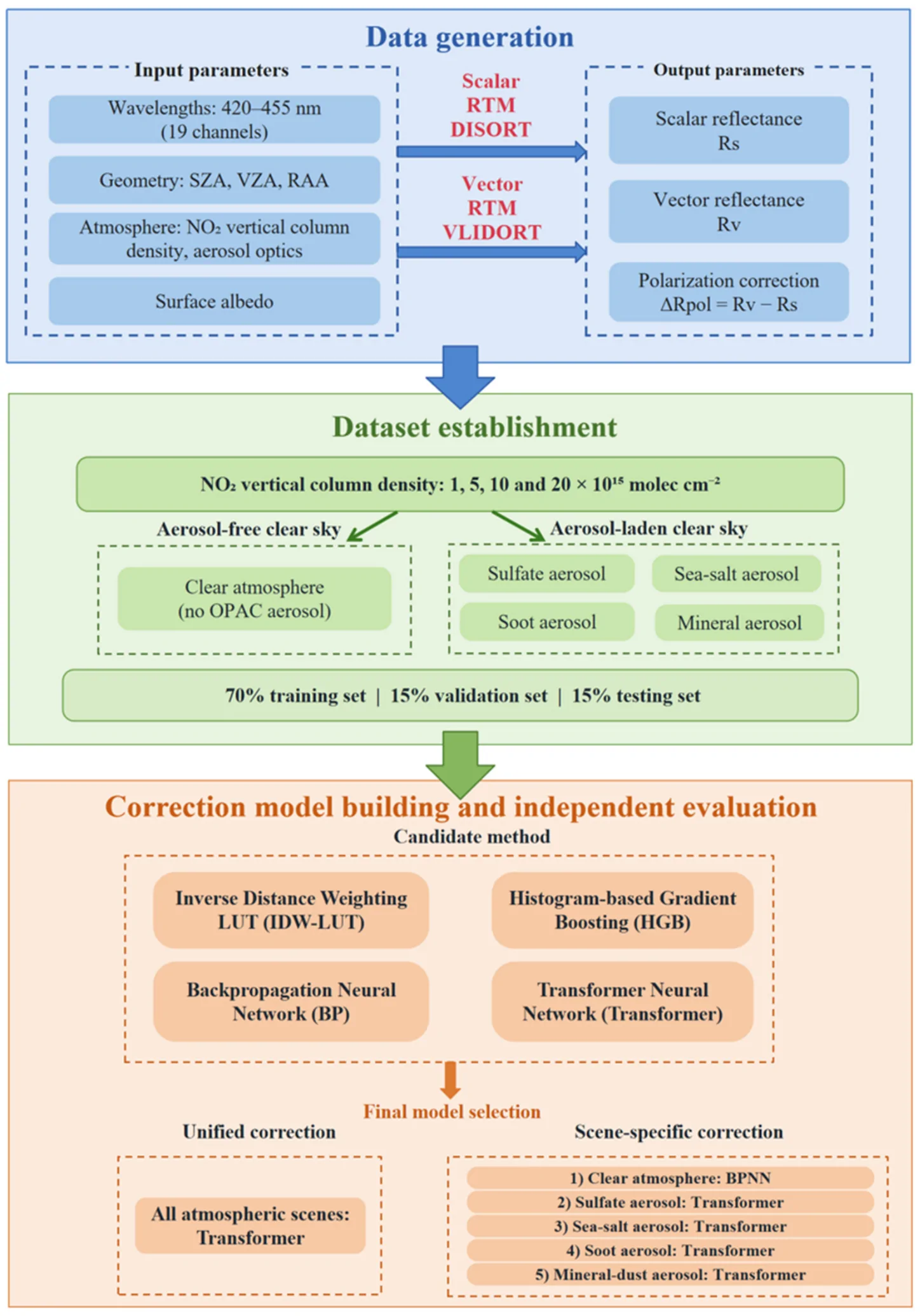
Training workflow adopted in this study. Arrows indicate the workflow direction, and background colors distinguish the three main stages.

Figure 1 outlines the workflow: paired scalar and vector radiative-transfer simulations are generated first, followed by data partitioning, correction-model training, and final model comparison. The analysis focuses on polarization correction in the 420–455 nm NO_2_ retrieval window. A scene-specific inverse-distance-weighted look-up table (IDW-LUT) provides the conventional baseline. Backpropagation neural network (BP), histogram-based gradient boosting (HGB), and Transformer models are trained either on all scenes together or separately within each scene.

### 2.1. Radiative Transfer Simulations

Paired top-of-atmosphere calculations were performed with the scalar radiative-transfer model DISORT and the vector model VLIDORT. Both calculations used the same atmospheric profiles, viewing geometry, surface conditions, and 32-stream discrete-ordinate configuration. VLIDORT retained the full Stokes vector and served as the reference, whereas DISORT neglected polarization. The simulations covered 19 wavelengths from 420 to 455 nm, with 2-nm spacing from 420 to 454 nm and an additional point at 455 nm. The atmosphere was represented by 40 layers spanning 0–80 km and included Rayleigh scattering and absorption by NO_2_, O_3_, and H_2_O. The atmosphere was cloud-free, the surface was Lambertian, and the surface pressure was fixed at 1013 hPa.

Four NO_2_ vertical column densities (VCD) were used: 1, 5, 10, and 20 × 10^15^ molecules cm^−2^. The solar zenith angle (SZA) and viewing zenith angle (VZA) each took values of 0°, 15°, 30°, 45°, 60°, and 75°. The relative azimuth angle (RAA), defined as the angular separation between the solar and viewing azimuth directions, was set to 0°, 60°, 90°, 120°, and 180°. Surface albedo was set to 0.02, 0.15, or 0.40. Five atmospheric scenes were simulated: one aerosol-free clear atmosphere and four aerosol scenes based on the Optical Properties of Aerosols and Clouds (OPAC) database [27], namely sulfate, sea salt, soot, and mineral dust. The aerosol optical properties included the wavelength-dependent extinction coefficient, single-scattering albedo, asymmetry factor, and phase-matrix expansion coefficients. Aerosols were uniformly distributed within the lowest 2 km of the atmosphere. Table 1 summarizes the radiative-transfer simulation setup.

The aerosol loading for each atmospheric scene was determined directly from the OPAC reference extinction coefficients, without normalization to a common aerosol optical depth (AOD). Each aerosol component was assumed to be uniformly distributed within the lowest 2 km of the atmosphere. Accordingly, the AOD at the reference wavelength of 440 nm was calculated as τ_a_(440) = β_ext_(440) × 2 km, where β_ext_(440) denotes the extinction coefficient at 440 nm. Table 2 summarizes the OPAC aerosol components, relative humidity, extinction coefficients, and corresponding AOD values for the five atmospheric scenes.

Radiance was converted to reflectance using $R\;=\;\frac{\pi I}{\mu_{0}}$, where I is the modeled top-of-atmosphere radiance normalized by the incident solar irradiance and μ_0_ is the cosine of the SZA. The scalar and vector reflectances are denoted by R_s_ and Rᵥ, respectively. Their difference defines the polarization correction, and each correction method estimates this difference before reconstructing the vector reflectance:

$${\Delta R}_{\mathrm{pol}}\;=\;R_{v}\;{-\;R}_{s},\;\hat{R_{v}}\;=\;R_{s}\;+\;\Delta \hat{R_{\mathrm{pol}}}.$$

Here, ${\Delta R}_{\mathrm{pol}}$ represents the scalar–vector reflectance difference primarily associated with the different treatment of polarization. The quantity $\Delta \hat{R_{\mathrm{pol}}}$ is the correction estimated by the IDW-LUT or machine-learning model, and $\hat{R_{v}}$ is the resulting estimate of the vector reflectance.

### 2.2. Dataset Construction and Partitioning

The full-factorial simulation grid comprised 205,200 spectral samples, corresponding to five atmospheric scenes, four NO_2_ VCD levels, 540 geometry–surface states, and 19 wavelengths. Each geometry–surface state was defined by a unique combination of SZA, VZA, RAA, and surface albedo. The 540 states were obtained from six SZA values, six VZA values, five RAA values, and three surface-albedo values.

Data partitioning was performed at the geometry–surface-state level. The 540 states were divided into 378 training states, 81 validation states, and 81 test states, corresponding to 70%, 15%, and 15%, respectively. All spectral samples belonging to the same state were retained in the same subset across the NO_2_ VCD levels and atmospheric scenes. This procedure prevented leakage of complete geometry–surface states across the data subsets. The input and output parameters of the correction methods are summarized in Table 3. The resulting training, validation, and testing subsets contained 143,640, 30,780, and 30,780 samples, respectively, with their distributions across the five atmospheric scenes summarized in Table 4. The testing set was used only after the model configurations and hyperparameters had been finalized.

### 2.3. Correction Models

Four correction approaches were evaluated using the same paired DISORT–VLIDORT database: a scene-specific inverse-distance-weighted lookup-table method (IDW-LUT), a backpropagation neural network (BP), histogram-based gradient boosting (HGB), and a Transformer neural network. All methods were designed to estimate the polarization correction between the scalar and vector radiative-transfer calculations, with the VLIDORT result serving as the reference. The IDW-LUT approach was implemented separately for each atmospheric scene, whereas the three machine-learning methods were evaluated using both unified and scene-specific training strategies.

As summarized in Table 3, the numerical inputs to the machine-learning models were NO_2_ VCD, wavelength, SZA, VZA, RAA, surface albedo, and scalar reflectance. The target variable was the polarization correction derived from the paired DISORT and VLIDORT simulations. For the unified models, aerosol type was included as an additional categorical predictor, using one-hot encoding for BP and HGB and a learned embedding for the Transformer. For the scene-specific models, aerosol type was used only to select the corresponding atmospheric-scene model and was not included as an input predictor. The same training, validation, and independent test partitions were used for all candidate models to ensure a consistent comparison.

All 19 simulated wavelengths were included in the training dataset, with wavelength treated as an explicit input feature. Each unified or scene-specific machine-learning model shared a single set of learned parameters across wavelengths rather than being optimized independently at individual wavelengths. However, the polarization corrections were predicted pointwise, and no explicit spectral smoothness constraint was imposed during training.

#### 2.3.1. Inverse Distance Weighted Lookup Table

Five scene-specific IDW-LUTs were constructed from the training samples, with separate LUTs developed for the clear atmosphere and for the sulfate, sea-salt, soot, and mineral-dust aerosol scenarios. Each LUT node represented one sampled combination of NO_2_ VCD, wavelength, SZA, VZA, RAA, and surface albedo, and stored the corresponding polarization correction derived from the paired scalar and vector simulations.

Before neighbor searching, the continuous LUT coordinates were independently normalized to [0, 1] using the training-set ranges to avoid scale differences in the distance calculation. Aerosol type was used only to select the corresponding scene-specific LUT, and scalar reflectance was excluded from the neighbor-search coordinates and used only for reconstructing the estimated vector reflectance.

For each query, the K nearest LUT nodes were identified using scipy.spatial.cKDTree [28,29] and weighted according to inverse distance [30]. Exact matches returned the stored correction directly. For queries that were not training nodes, an inverse-distance-weighted local first-order regression with ridge regularization was fitted to the neighboring corrections to estimate the polarization correction [31]. The estimated correction was then added to the scalar reflectance to reconstruct the vector-equivalent reflectance.

The neighborhood size K, the inverse-distance exponent *p*, and the ridge coefficient α were tuned separately for each atmospheric scene by minimizing the validation-set relative root-mean-square error (RRMSE). The selected distance exponent was *p* = 3 for all five scenes. The clear-atmosphere, sulfate, and mineral-dust LUTs used K = 64 and α = 10^−8^; the soot LUT used K = 64 and α = 10^−10^; and the sea-salt LUT used K = 32 and α = 10^−4^. These choices were determined using the validation subset only, and the independent testing subset was not involved in LUT hyperparameter selection.

#### 2.3.2. Backpropagation Neural Network (BP)

BP was used as a fully connected feed-forward regression model. Backpropagation updates network parameters by propagating prediction errors from the output layer toward earlier layers and remains a standard approach for learning nonlinear mappings from multivariate inputs [32]. Neural-network emulators have also been used in atmospheric remote sensing to approximate expensive radiative-transfer calculations and to correct simplified forward models [17,18].

The BP architecture used three hidden layers containing 256, 128, and 64 neurons. Gaussian Error Linear Unit (GELU) activation was applied between hidden layers, and a dropout probability of 0.03 was used during training to reduce overfitting. Optimization was performed with AdamW, which applies weight decay separately from the gradient-based parameter update. The initial learning rate, weight-decay coefficient, mini-batch size, maximum number of epochs, early-stopping patience, and gradient-clipping threshold were set according to the hyperparameter configuration provided in Table 5.

During training, model performance was monitored on the validation subset after each epoch. Early stopping was used to terminate optimization when validation performance ceased to improve for the prescribed patience interval, and the model state corresponding to the best validation performance was retained for subsequent testing. Gradient-norm clipping was applied to reduce the risk of unstable parameter updates. The unified and scene-specific BP models used the same architecture and optimization settings; their difference was the composition of the training data and the treatment of aerosol type.

#### 2.3.3. Histogram-Based Gradient Boosting (HGB)

HGB was included as a tree-based nonlinear regression approach with a modeling structure that differs substantially from neural networks. Gradient boosting builds an additive ensemble sequentially, with each new regression tree focusing on residual structure that is not adequately represented by the preceding ensemble [33]. Histogram-based implementations improve computational efficiency by grouping continuous feature values into discrete bins before evaluating candidate split points, which is advantageous for relatively large tabular datasets such as the paired radiative-transfer database used here.

The HGB configuration used a learning rate of 0.04 and a maximum of 4000 boosting iterations. Individual trees were limited to 31 leaf nodes, with at least 20 samples required in each leaf, while no explicit maximum tree depth was imposed. A maximum of 255 histogram bins and an L2 regularization coefficient of 10^−6^ were used, and the full feature set was available at each boosting stage. The regression objective was squared error, and early stopping was disabled so that both unified and scene-specific models were trained under the same fixed boosting budget. The complete HGB settings are listed in Table 5.

#### 2.3.4. Transformer Neural Network

The Transformer was selected as an attention-based neural-network architecture for representing interactions among the structured numerical and categorical predictors used in polarization correction [34]. The scalar-vector reflectance difference depends on coupled nonlinear relationships involving wavelength, NO_2_ vertical column density, viewing geometry, surface albedo, scalar reflectance, and aerosol properties. We therefore evaluated whether an attention-based architecture could effectively represent these feature interactions, alongside the fully connected BP network and the tree-based HGB model. The inclusion of these three model families was intended to examine different regression approaches under a common evaluation framework. In this application, the physical, geometric, spectral, surface, and scalar-reflectance predictors were represented within a common embedding space before being passed through Transformer encoder layers. For the unified model, aerosol type was represented by a learned categorical embedding so that atmospheric-scene information could be incorporated jointly with the continuous predictors. This categorical input was omitted in the scene-specific models because the scene identity was already fixed by model selection.

The Transformer used an embedding dimension of 32, four attention heads, two encoder layers, and a feed-forward hidden dimension of 128. GELU activation and a dropout probability of 0.05 were used within the network. Training was performed with AdamW. The learning rate, weight decay, mini-batch size, maximum number of epochs, early-stopping patience, and gradient-clipping threshold followed the settings reported in Table 5. As with BP, validation performance was monitored during training, and the checkpoint with the best validation performance was retained for independent evaluation.

### 2.4. Evaluation Measures

For test sample i, the signed relative error was calculated from the reference vector reflectance R_v,i_ and the scalar or corrected estimate R_m,i_:

$$e_{i}\;=\;100\frac{R_{m,i}\;-\;R_{v,i}}{R_{v,i}}.$$

Samples with |R_v,i_| ≤ 10^−8^ were excluded from relative-error calculations; no test sample met this exclusion criterion. Accuracy was summarized by the mean absolute relative error (MARE), signed bias, relative root-mean-square error (RRMSE), and the 95th percentile of |e_i_| (P95):

$$\mathrm{MARE}\;=\;\frac{1}{N}\sum_{i=1}^{N}\left|e_{i}\right|,\;\mathrm{Bias}\;=\;\frac{1}{N}\sum_{i=1}^{N}e_{i},$$

$$\mathrm{RRMSE}=\sqrt{\frac{1}{N}\sum_{i=1}^{N}e_{i}^{2}},\;P95={\;\mathrm{percentile}}_{95}\left(\left|e_{i}\right|\right).$$

The reduction in RRMSE relative to the scalar baseline quantified the fraction of the original scalar error removed by each correction method:

$$\mathrm{RRMSE}\;\mathrm{reduction}\;=\;100\left(1\;-\;\frac{{\mathrm{RRMSE}}_{\mathrm{method}}}{{\mathrm{RRMSE}}_{\mathrm{scalar}}}\right).$$

The scalar baseline used R_s_ directly. Overall values were calculated from the combined test samples of all five scenes, and scene-wise values were calculated separately to assess how unified and scene-specific training behaved under each aerosol condition.

To further evaluate local wavelength-to-wavelength fluctuations in the correction residuals, we introduced a spectral residual roughness metric (Q), defined as the root-mean-square second-order difference of the signed relative reflectance errors along the wavelength dimension:

$$Q=\sqrt{\frac{1}{N_{\lambda }-2}\sum_{j=2}^{N_{\lambda }-1}{\left(e_{j+1}-2e_{j}+e_{j-1}\right)}^{2}}.$$

where e_j_ denotes the signed relative reflectance error at the j-th wavelength of a given complete test spectrum, calculated using the relative-error definition of e_i_ provided above. The subscript j indexes the wavelength within a complete spectrum, whereas i in the preceding definition indexes individual test samples. N_λ_ denotes the number of uniformly spaced wavelength samples included in the roughness calculation; the summation runs over the interior wavelength indices.

For each complete test spectrum, Q was calculated using 18 uniformly spaced wavelengths from 420 to 454 nm (N_λ_ = 18; wavelength spacing = 2 nm). The final wavelength at 455 nm was excluded because its separation from the preceding point is only 1 nm. Since e_j_ is expressed in percent, Q is reported in percentage points. Lower Q values indicate weaker local wavelength-to-wavelength fluctuations in the spectral residuals.

## 3. Results

Based on the paired scalar–vector simulation database, data partitioning strategy, and evaluation measures described in Section 2, the results are presented in two stages. First, we examine the magnitude and physical structure of the scalar–vector reflectance difference across wavelength, NO_2_ VCD, viewing geometry, surface albedo, and atmospheric scene. The correction methods are then evaluated on the independent test set in terms of error distribution, overall and scene-wise accuracy, computational time, and storage requirements.

**Figure 2:**
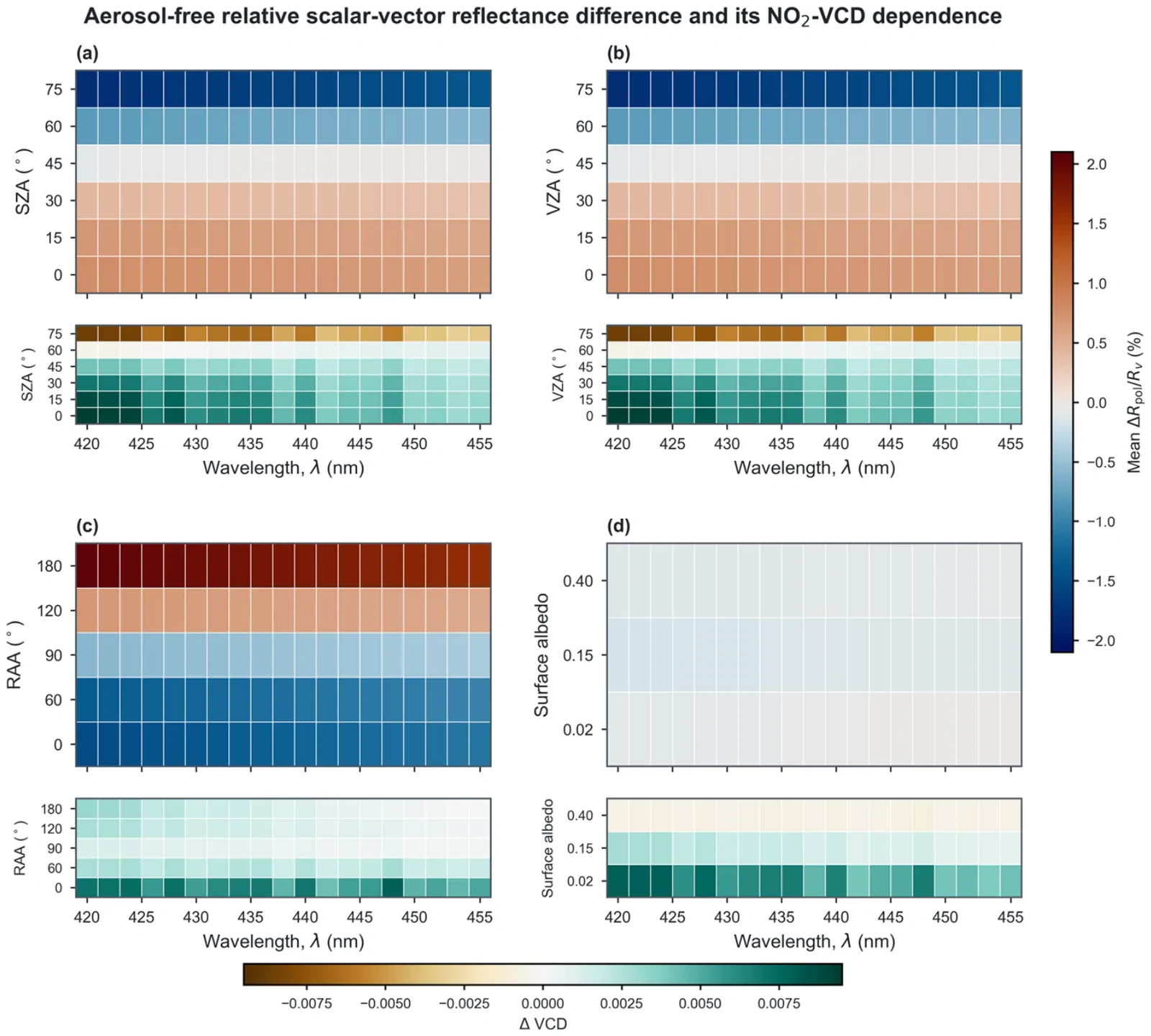
Aerosol-free relative scalar–vector reflectance difference and its NO_2_-VCD dependence as functions of wavelength and (**a**) SZA, (**b**) VZA, (**c**) RAA, and (**d**) surface albedo. The upper heat maps show the mean 100ΔR_pol_/R_v_, where ΔR_pol_ = (R_v_ − R_s_), averaged over the remaining scene variables and four NO_2_ VCD levels. The lower heat maps show the factor-specific ΔVCD, calculated as the mean 100ΔR_pol_/R_v_ at an NO_2_ VCD of 20 × 10^15^ minus the corresponding mean at 1 × 10^15^ molecules cm^−2^, after averaging over the remaining scene variables.

The scale and structure of the correction in the aerosol-free case is shown in Figure 2. The scalar–vector difference changes with wavelength and viewing geometry rather than behaving as a constant offset. The largest relative differences occur for oblique illumination or viewing, where photon paths are longer and multiple scattering is more important. The lower maps also show a dependence on NO_2_ VCD: the correction at 20 × 10^15^ molecules cm^−2^ differs from that at 1 × 10^15^ molecules cm^−2^. Wavelength and VCD were therefore retained as explicit predictors instead of using a single wavelength-independent adjustment.

**Figure 3:**
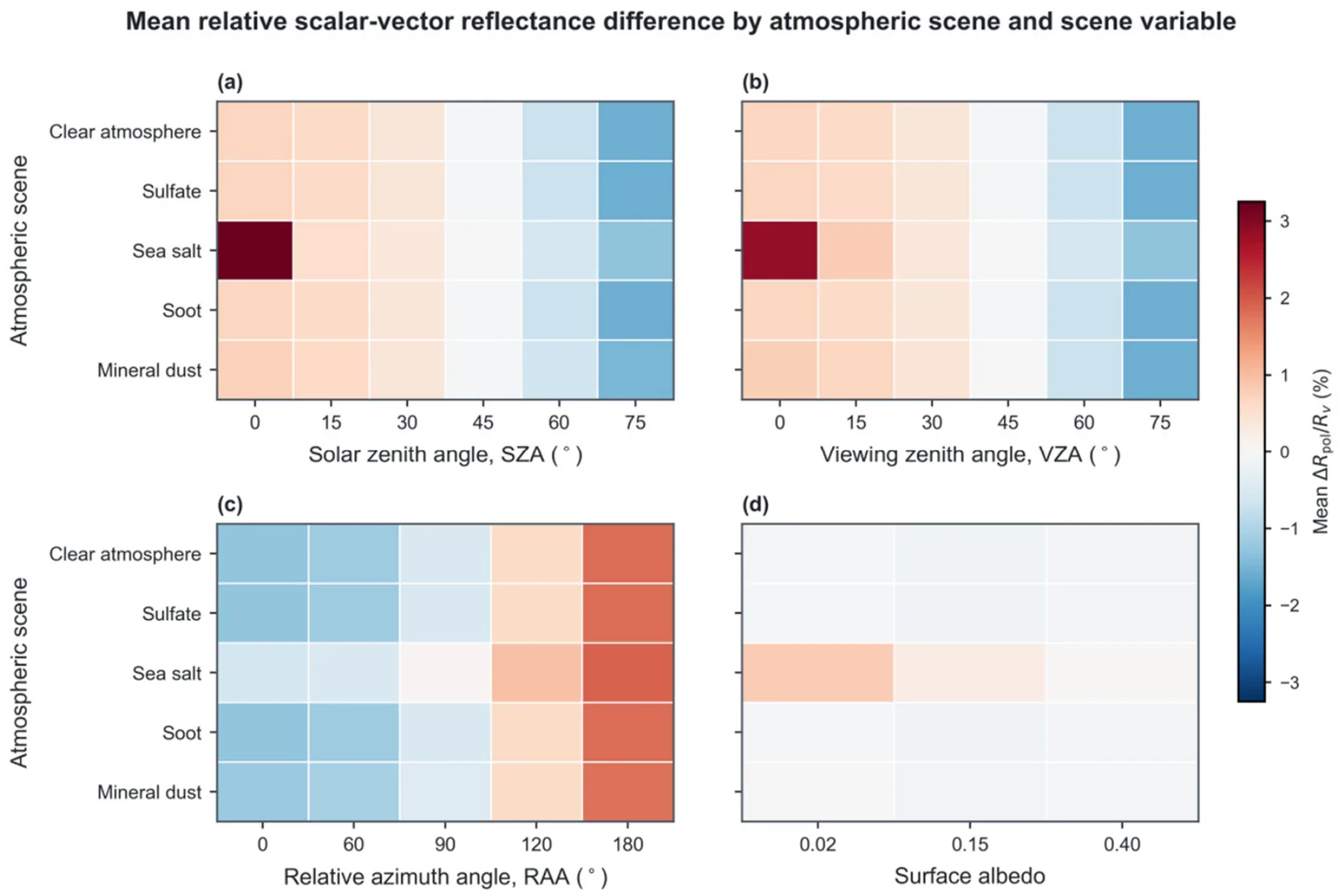
Mean relative scalar–vector reflectance difference 100ΔR_pol_/R_v_, where ΔR_pol_ = (R_v_ − R_s_) for five atmospheric scenes as functions of (**a**) SZA, (**b**) VZA, (**c**) RAA, and (**d**) surface albedo. Values are averaged over all remaining variables.

Figure 3 extends the aerosol-free analysis in Figure 2 by comparing the mean scalar-vector reflectance difference among the five atmospheric scenes. The correction retains a broadly consistent angular structure across scenes: it changes from positive to negative as SZA or VZA increases, whereas increasing RAA produces the opposite transition. Surface albedo has a comparatively weak effect. Aerosol type mainly modifies the magnitude of these patterns rather than their overall direction. Sea salt shows the clearest departure, with a particularly large positive difference at low SZA and VZA and a stronger albedo dependence than the other scenes. These scene-dependent responses indicate that polarization correction must account for interactions between viewing geometry and aerosol properties.

**Figure 4:**
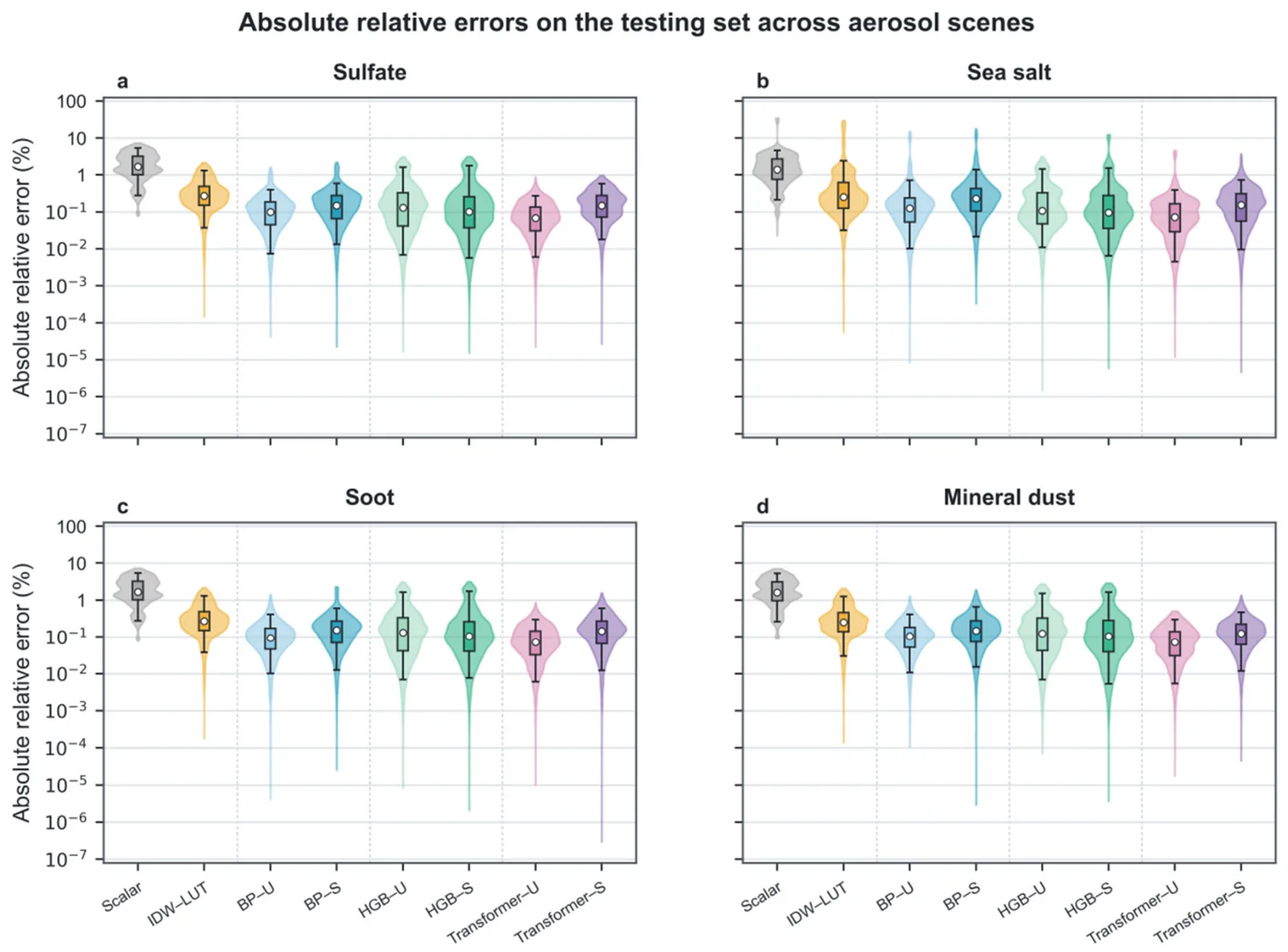
Distributions of the absolute relative reflectance error, |100(R_method_ − R_v_)/R_v_| on the testing set for (**a**) sulfate, (**b**) sea-salt, (**c**) soot, and (**d**) mineral-dust aerosol scenes. U and S denote unified and scene-specific models, respectively. The vertical axis is logarithmic.

The error distributions on the independent test set for the four aerosol scenes are summarized in Figure 4. All correction families narrow the central distribution and shorten the long tails relative to the scalar calculation, but the degree of improvement depends on both method and scene. The unified Transformer usually gives the narrowest distributions, followed by unified BP and HGB; scene-specific models are not consistently better. The logarithmic ordinate makes the rare large errors visible. Sea salt retains a broader tail for several methods, in line with the stronger angular sensitivity seen in Figure 3.

**Figure 5:**
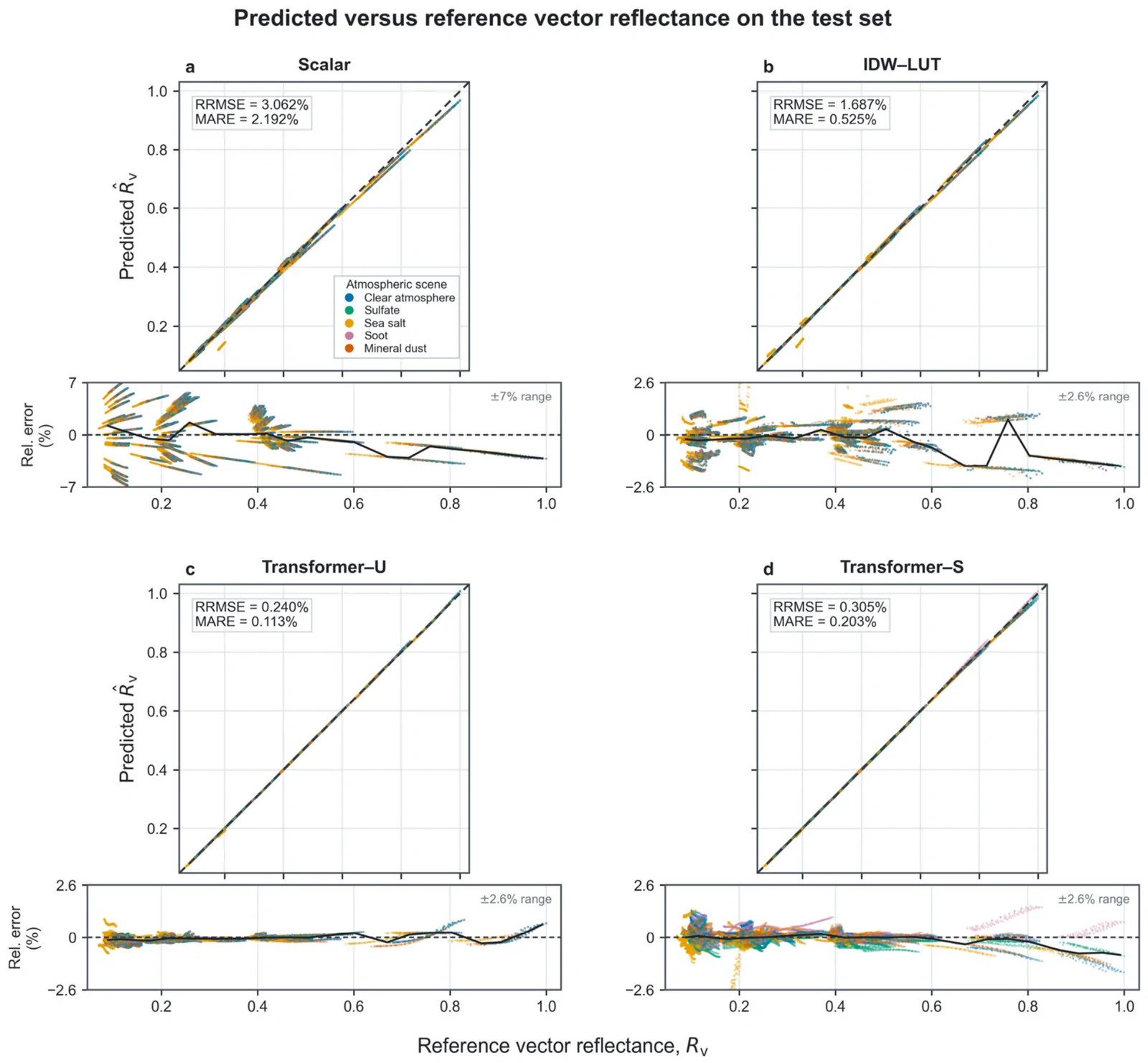
Predicted versus reference vector reflectance and corresponding relative errors on the testing set for (**a**) the scalar baseline, (**b**) IDW-LUT, (**c**,**d**) Transformer. U and S denote unified and scene-specific models, respectively. The dashed diagonal lines in the upper panels represent the 1:1 reference line, while the dashed horizontal lines in the lower panels indicate zero relative error. The black solid lines show median relative errors calculated in 20 equal-width reference-reflectance bins. The lower panels display the central 99% of relative errors.

Figure 5 compares the predicted and reference reflectances over the full reflectance range. The scalar baseline departs from the one-to-one relation and shows a reference-dependent error pattern. IDW-LUT removes much of the systematic displacement but retains local scatter between sampled nodes. The Transformer predictions lie close to the one-to-one line, and their binned median relative errors remain near zero over most of the range. The scene-specific Transformer is also well calibrated, although its wider spread in some bins indicates that specialization does not automatically improve generalization when fewer training states are available.

Table 6 reports the combined results for the 30,780 independent test samples. The scalar baseline has an RRMSE of 3.0620% and a MARE of 2.1916%. IDW-LUT removes 44.90% of the scalar RRMSE, reducing it to 1.6872%, although its residual bias remains −0.1717%. Unified BP and HGB reduce the RRMSE to 0.6282% and 0.5430%, respectively. The unified Transformer performs best, with MARE = 0.1133%, bias = −0.0564%, RRMSE = 0.2402%, and P95 = 0.3008%, corresponding to a 92.15% reduction from the scalar baseline. The scene-specific Transformer is only slightly less accurate (RRMSE = 0.3050%), while the scene-specific BP and HGB models are weaker than their unified counterparts. For this balanced set of scenes, sharing the mapping and providing aerosol type as an input works better than dividing the data among five smaller models.

The scene-wise results in Table 7 confirm that the unified Transformer remains strong across all five scenes, with RRMSE values of 0.1372–0.4557% and reductions of 89.01–95.01%. Its lowest errors occur for sulfate and mineral dust, whereas sea salt is the most difficult scene. For the unified Transformer, the sea-salt scene yielded the highest RRMSE (0.4557%). This was also the only atmospheric scene in which the scene-specific Transformer achieved a lower RRMSE than the unified Transformer (0.4009% versus 0.4557%). In the other four atmospheric scenes, the unified Transformer performed better, indicating that combining aerosol scenes did not systematically degrade correction accuracy, although scene-dependent compromises may occur. Unified BP is competitive for soot and mineral dust, but its sea-salt RRMSE reaches 1.3349%. HGB is more stable than BP in some aerosol cases, although its larger P95 values indicate heavier tails. Scene-specific training improves a few HGB entries, but the gains are not systematic; in this factorial design, the loss of training diversity offsets the potential benefit of specialization.

These scene-dependent differences indicate that polarization correction performance is sensitive to the aerosol conditions represented in the simulations. In particular, the sea-salt scene exhibits both stronger angular variations in the scalar-vector reflectance difference (Figure 3) and larger residual correction errors (Table 7). Nevertheless, the unified Transformer substantially reduces the scalar reflectance errors across all four aerosol scenes, demonstrating its effectiveness under the specific aerosol conditions considered in this study.

**Figure 6:**
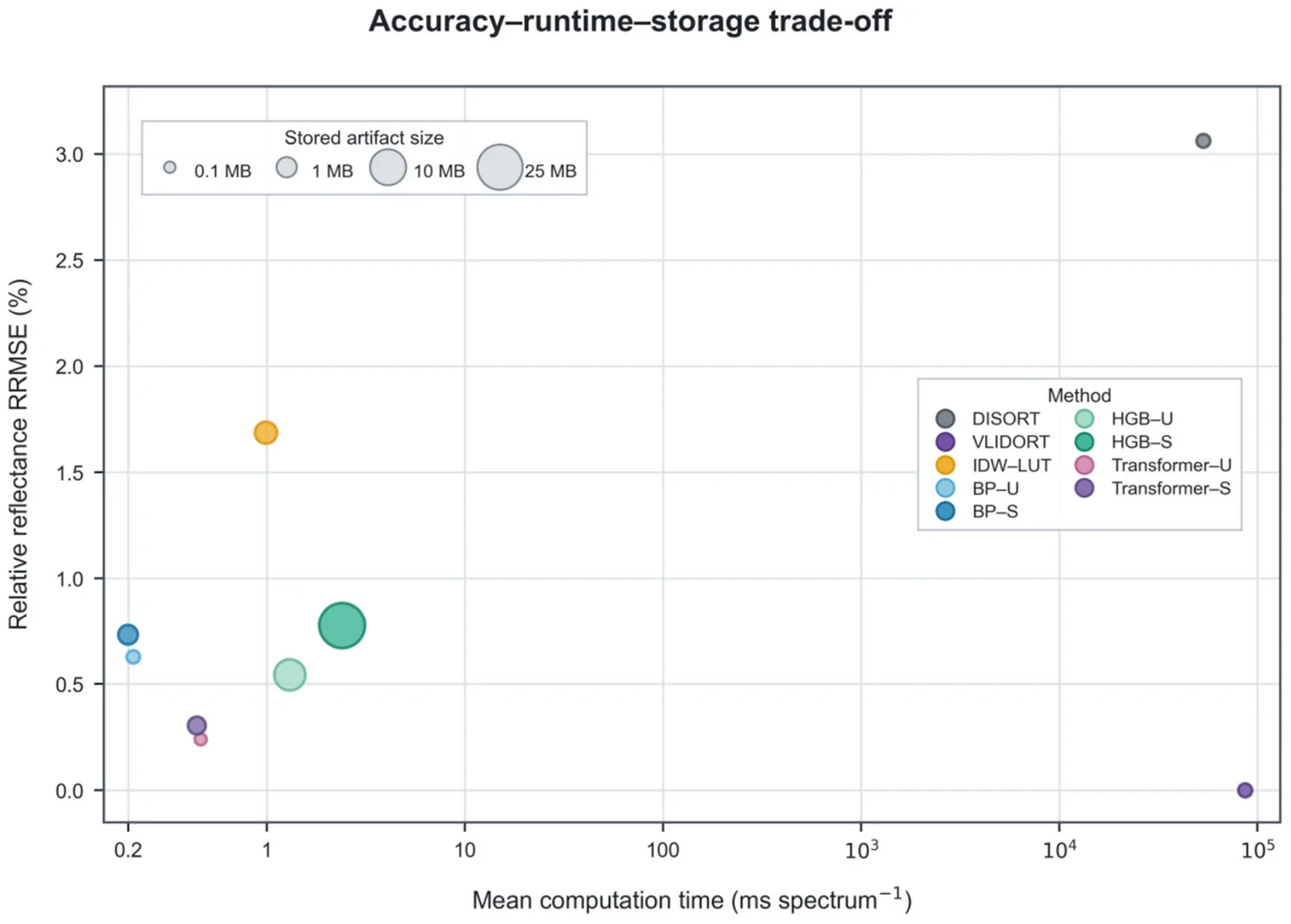
Accuracy–runtime–storage trade-off of radiative-transfer and correction methods. The *x*-axis shows the mean computation time per 19-wavelength spectrum (log scale), the *y*-axis shows test-set RRMSE relative to VLIDORT, and bubble area represents stored model or LUT size. U and S denote unified and scene-specific models, respectively.

Table 8 and Figure 6 put the accuracy results alongside the computational costs. VLIDORT requires approximately 8.7 × 10^4^ ms per 19-wavelength spectrum, compared with 5.3 × 10^4^ ms for DISORT. The correction times reported for IDW-LUT and the ML models are incremental costs after the scalar reflectance is available: IDW-LUT requires 0.9892 ms per spectrum and stores 1.39 MiB; the unified Transformer requires 0.4636 ms and stores 0.1159 MiB; and unified BP has the lowest ML latency at 0.2115 ms. The unified Transformer has the largest offline training time among the unified models (1442.75 s), whereas HGB has the largest unified artifact (5.4852 MiB). Scene-specific variants add storage and, for HGB, online cost without a consistent accuracy benefit. On this incremental-cost basis, the unified Transformer provides the best accuracy–storage balance and unified BP is the low-latency option.

**Figure 7:**
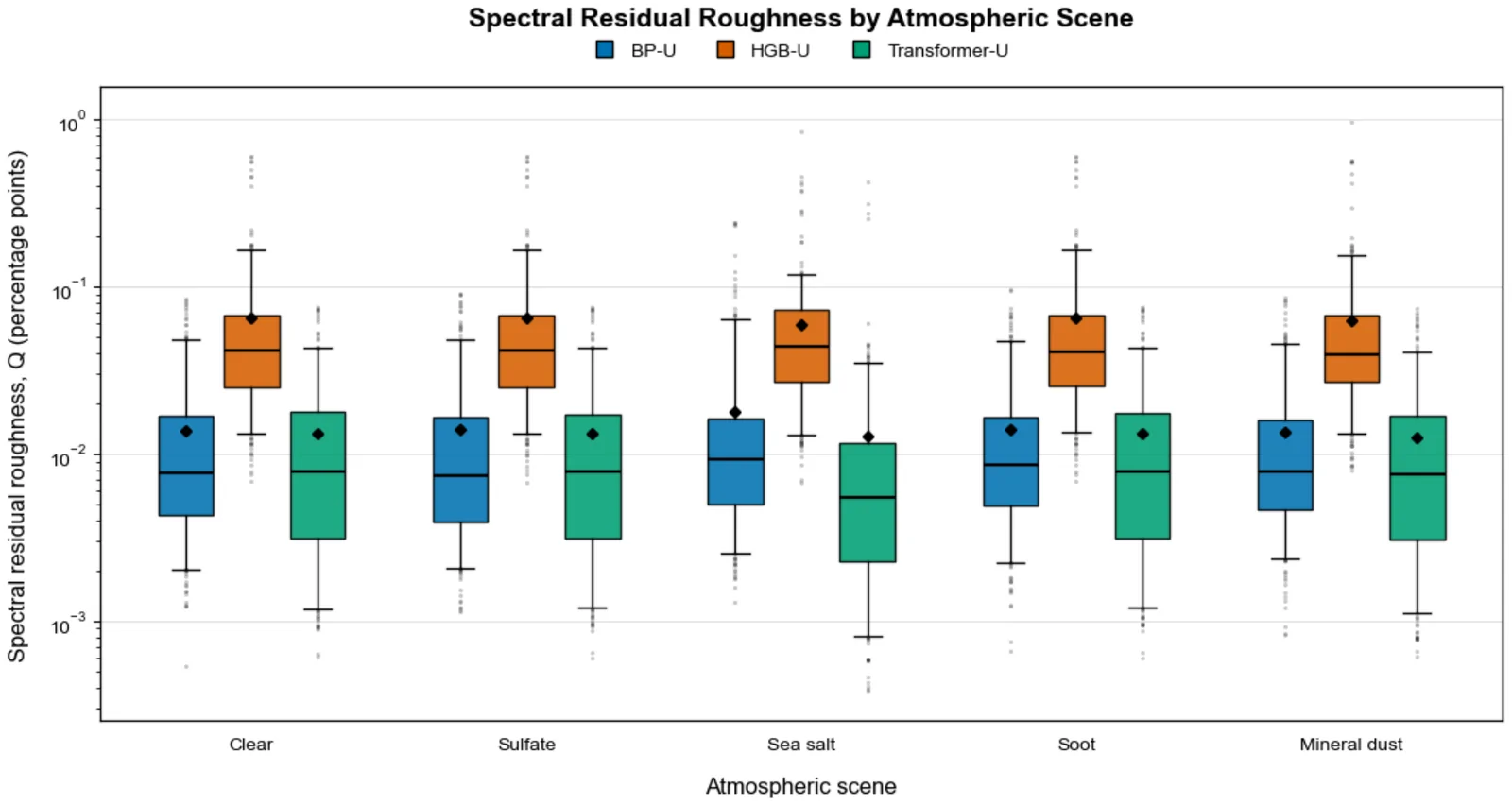
Distributions of spectral residual roughness (Q) for the unified BP, HGB, and Transformer correction models across five atmospheric scenes. Black diamonds indicate the means, and horizontal lines within the boxes indicate the medians. Lower Q values indicate weaker local wavelength-to-wavelength fluctuations in the spectral residuals.

To further assess the spectral consistency of the correction models, Figure 7 presents the distributions of spectral residual roughness across the five atmospheric scenes. HGB-U exhibits substantially higher roughness than BP-U and Transformer-U, indicating stronger local wavelength-to-wavelength fluctuations in its residual errors. In contrast, Transformer-U maintains relatively low roughness across all atmospheric scenes, with distributions broadly comparable to those of BP-U. Representative spectral comparisons are provided in Supplementary Figures S1 and S2. Figure S1 illustrates a selected clear-atmosphere case, whereas Figure S2 presents a challenging sea-salt case identified by the maximum absolute local residual curvature within the sea-salt test subset. In the latter case, HGB exhibits pronounced local fluctuations in its predicted corrections, whereas the Transformer predictions follow the VLIDORT reference spectral trend more smoothly. Combined with its lower overall RRMSE, these results indicate that the unified Transformer provides a favorable balance between pointwise correction accuracy and spectral residual consistency.

To further evaluate the sensitivity of the unified Transformer to missing input data, additional experiments were conducted using the pretrained model without updating its parameters. Synthetic missingness was introduced into five numerical predictors (NO_2_ VCD, SZA, VZA, RAA, and surface albedo), with missing values replaced by their corresponding training-set means. Further details of the missingness generation and training-mean imputation procedures are provided in Supplementary Note S1. Wavelength, scalar reflectance, and aerosol identity remained available. The overall RRMSE increased from 0.2402% with complete inputs to 1.2242% ± 0.1896% and 2.0731% ± 0.1467% at nominal missingness probabilities of 10% and 30%, respectively (Figure S3 and Table S1). When individual angular predictors were unavailable for all test samples, the RRMSE increased to 2.4467% for SZA, 2.4773% for VZA, and 1.8550% for RAA (Figure S4 and Table S2). These results indicate that the unified Transformer is particularly sensitive to the availability of angular information under the specified training-mean imputation procedure.

## 4. Discussion

The results above indicate that the DISORT-VLIDORT scalar-to-vector correction target exhibits systematic physical structure rather than random numerical variation. Wavelength, VCD, geometry, surface albedo, and aerosol optical properties jointly determine ΔR_pol_, so a single empirical offset cannot meet a sub-percent target across the full domain. The test comparison also shows that model capacity and scene handling need to be considered together. Because the unified Transformer sees all balanced scenes while retaining aerosol identity through a categorical embedding, it can represent shared behavior and scene-dependent interactions without dividing the database into smaller parts.

Scene-specific training remains useful for diagnosis and may be appropriate when the operational scene distribution is narrow and aerosol type is known with high confidence. It did not improve the average result here because each scene-specific model saw only one-fifth of the atmospheric diversity. Nevertheless, the slightly better performance of the scene-specific Transformer in the sea-salt scene suggests that shared model parameters may involve scene-dependent trade-offs, even when aerosol identity is explicitly represented. Sea salt provides the clearest stress test: its larger residual tails persist across several models, suggesting that a better representation of aerosol phase behavior or denser sampling in the relevant angular region may be more useful than simply increasing network size.

Physically, aerosol type affects the absorption characteristics, single-scattering albedo, and scattering phase-matrix properties, whereas aerosol loading modifies the atmospheric optical thickness and the relative contributions of aerosol and molecular scattering. Both factors can therefore influence the magnitude and angular dependence of the scalar-vector reflectance difference and, consequently, the performance of the polarization correction. However, in the present study, only one aerosol loading level was simulated for each aerosol type, characterized by its AOD at the reference wavelength of 440 nm. At the other simulated wavelengths, AOD varied according to the wavelength-dependent OPAC extinction coefficients. The aerosol scenes were not normalized to the same AOD, and aerosol loading was not varied independently within each aerosol type. Therefore, the observed differences among aerosol scenes reflect the combined effects of aerosol type and loading, and their individual contributions cannot be separated using the present experimental design. The present scene-wise comparison should consequently be interpreted as a comparison among the prescribed OPAC aerosol scenarios, rather than as a controlled isolation of aerosol-type effects.

Hygroscopic growth may further influence polarization correction by modifying aerosol particle size and scattering phase-matrix properties, particularly for hygroscopic aerosols such as sea salt and sulfate [27,35]. In the present simulations, both components were represented using OPAC optical properties at a fixed RH of 50%. Although the sea-salt scene exhibited larger residual correction errors, the contribution of hygroscopic growth cannot be isolated because relative humidity was not varied independently. Therefore, the sensitivity of polarization correction to humidity-induced changes in aerosol optical properties requires further investigation.

The correction should therefore be regarded as valid only within the simulated domain: wavelengths of 420–455 nm, NO_2_ VCDs of 1–20 × 10^15^ molecules cm^−2^, SZA and VZA values of 0–75°, RAA values of 0–180°, surface albedos of 0.02–0.40, and cloud-free atmospheres containing one of the pure, known aerosol scenes at the prescribed aerosol loading levels. Atmospheric states outside the simulated conditions, including different aerosol loadings and mixed or unidentified aerosol conditions, should be treated as extrapolation cases requiring additional validation.

The timing results separate offline preparation from online inference. Transformer training is relatively expensive, but it is performed once; retrieval processing repeatedly incurs only the per-spectrum inference cost. Unified BP is attractive when latency dominates, whereas the unified Transformer is preferable when the accuracy requirement is stricter and a sub-megabyte artifact is desired. IDW-LUT remains transparent and easy to validate, but its interpolation error and larger storage footprint limit its performance on held-out geometry–surface states. These comparisons depend on the stated hardware, implementation, and 19-wavelength spectrum and should not be treated as universal benchmarks across software stacks.

Several limitations define the scope of the conclusions. The simulations are cloud-free, use one standard atmospheric profile, place each aerosol type uniformly in the lowest 2 km, and consider only pure, known aerosol scenes. The geometry and surface grids are deliberately controlled, and the test partition holds out complete geometry–surface states rather than representing every possible natural atmosphere. The model comparison was limited to BP, HGB, and Transformer architectures and should not be interpreted as a comprehensive benchmark of tabular-learning methods. Additional comparisons with model families such as XGBoost, LightGBM, and TabNet, using model-specific hyperparameter optimization and the same independent evaluation protocol, are required before broader conclusions regarding architecture superiority can be drawn. Although the machine-learning models share learned parameters across wavelengths, no explicit spectral smoothness constraint was imposed during training. The spectral residual roughness analysis quantifies local wavelength-to-wavelength fluctuations at the simulated sampling intervals but cannot rule out non-physical spectral oscillations at finer spectral resolutions. More generally, although the learning targets were derived from physically based radiative-transfer simulations, the current data-fitting objectives did not explicitly enforce physical constraints, and the possibility of learning spurious relationships among input variables cannot be entirely excluded. The additional missing-input experiments further indicate that the pretrained Transformer can exhibit substantial performance degradation when important numerical predictors are unavailable, particularly those describing viewing geometry. However, these results characterize the frozen model under training-mean imputation rather than a model explicitly trained to handle missing values, and its robustness under other missing-data conditions remains to be evaluated.

An additional limitation concerns the attribution of the DISORT–VLIDORT reflectance difference. Although DISORT and VLIDORT were configured with identical atmospheric, geometric, surface, and 32-stream settings, a matched comparison between DISORT and VLIDORT operated in scalar mode (NSTOKES = 1) was not performed in the present study. Consequently, the correction target may contain a solver-dependent contribution in addition to the effect of neglecting polarization. The VLIDORT 2.8.1 User’s Guide identifies scalar-mode comparisons with an independent scalar solver as a fundamental validation test. Moreover, Escribano et al. [36] reported a mean fractional difference below 0.1% between DISORT and scalar-mode VLIDORT for 32-stream calculations over Lambertian surfaces. This published result suggests that solver-dependent differences are likely small for configurations similar to ours. However, because their wavelengths, atmospheric profiles, aerosol conditions, and angular domain are not identical to those used here, the benchmark does not provide a direct quantitative validation for the present simulations. A matched DISORT–scalar-VLIDORT comparison over the full simulation domain is therefore required in future work to isolate the polarization contribution more rigorously.

Moreover, the reported metrics quantify only the accuracy of the scalar-to-vector correction at the reflectance level; they do not by themselves establish the corresponding uncertainty in retrieved NO_2_ slant or vertical columns. Finally, this study evaluates reflectance correction only. Propagation of the remaining spectral residual into NO_2_ slant-column or vertical-column error depends on the fitting algorithm, absorption cross sections, instrument line shape and noise, and polynomial or wavelength-calibration terms; that retrieval-level uncertainty is outside the present experiment. Future work should therefore include mixed aerosols, variable vertical distributions, clouds, realistic instrument convolution, and an end-to-end NO_2_ fitting assessment, as well as the development and evaluation of physics-informed regularization strategies that preserve physically meaningful NO_2_ absorption features.

## 5. Conclusions

This study developed and evaluated unified and scene-specific corrections from scalar DISORT reflectances to vector VLIDORT Stokes-I reflectances in the 420–455 nm visible NO_2_ retrieval window. Across 205,200 paired spectral samples from an aerosol-free scene and four pure aerosol scenes, the scalar–vector difference varied systematically with wavelength, NO_2_ VCD, viewing geometry, surface albedo, and aerosol optical properties.

On the independent test set, the uncorrected scalar baseline reached RRMSE = 3.0620%. IDW-LUT reduced this error by 44.90%, while unified BP and HGB achieved reductions of 79.48% and 82.27%. The unified Transformer gave the lowest error, with MARE = 0.1133%, RRMSE = 0.2402%, P95 = 0.3008%, and a 92.15% RRMSE reduction. It achieved substantial error reductions across all five scenes, although the residual error was largest for sea salt.

The efficiency results point to two practical choices: unified Transformer when accuracy and storage are most important, and unified BP when online latency is the priority. These conclusions apply to the clear-sky, single-aerosol, known-scene conditions represented here. Mixed aerosols, clouds, variable aerosol profiles, instrument effects, and end-to-end NO_2_ column retrieval remain to be tested before operational use.

## Figures and Tables

**Table 1:** Spectral, atmospheric, geometric, surface, and aerosol settings used in the radiative-transfer simulations.

Parameter	Setting
Spectral window	420–454 nm at 2 nm intervals, plus 455 nm (19 wavelengths)
NO_2_ vertical column density	1, 5, 10 and 20 × 10^15^ molecules cm^−2^
Surface pressure	1013 hPa
Cloud condition	Cloud-free
Solar zenith angle (SZA)	0°, 15°, 30°, 45°, 60° and 75°
Viewing zenith angle (VZA)	0°, 15°, 30°, 45°, 60° and 75°
Relative azimuth angle (RAA)	0°, 60°, 90°, 120° and 180°
Surface albedo	0.02, 0.15 and 0.40
Aerosol scenes	Clear, sulfate, sea salt, soot and mineral dust
Aerosol optical properties	Extinction, SSA, asymmetry factor and phase matrix
Number of streams	32

**Table 2:** OPAC aerosol components, relative humidity, extinction coefficients, and AOD at 440 nm.

Atmospheric Scene	OPAC Component	RH (%)	Extinction Coefficient at 440 nm (km^−1^)	AOD at 440 nm
Clear atmosphere	/	/	0	0
Sulfate	Water-soluble sulfate (suso50)	50	1.9082 × 10^−4^	3.8164 × 10^−4^
Sea salt	Coarse-mode sea salt (sscm50)	50	1.4290 × 10^−1^	2.8580 × 10^−1^
Soot	Soot (soot00)	0	7.6224 × 10^−7^	1.5245 × 10^−6^
Mineral dust	Accumulation-mode mineral dust (mian00)	0	3.0302 × 10^−3^	6.0604 × 10^−3^

Note: The OPAC reference extinction coefficients were applied directly. Each aerosol component was uniformly assigned to the lowest 2-km atmospheric layer; therefore, the AOD at 440 nm was calculated as τ_a_(440) = β_ext_(440) × 2 km. The aerosol scenes were not normalized to the same AOD.

**Table 3:** Input and output parameters of the IDW-LUT, unified ML, and scene-specific ML correction methods.

Parameter Set	IDW-LUT	Unified ML	Scene-Specific ML
Input parameter	NO_2_ VCD (molecules cm^−2^)	NO_2_ VCD (molecules cm^−2^)	NO_2_ VCD (molecules cm^−2^)
	Wavelength (nm)	Wavelength (nm)	Wavelength (nm)
	SZA (°)	SZA (°)	SZA (°)
	VZA (°)	VZA (°)	VZA (°)
	RAA (°)	RAA (°)	RAA (°)
	Surface albedo	Surface albedo	Surface albedo
	Aerosol type (used to select the corresponding scene-specific LUT; excluded from distance calculation and interpolation)	Aerosol type (categorical model input: one-hot encoded for BP/HGB and embedded for Transformer)	Aerosol type (used to select the corresponding scene-specific model; not included as a model input)
	Scalar reflectance, R_s_	Scalar reflectance, R_s_	Scalar reflectance, R_s_
Output parameter	Interpolated correction, ΔR_pol_	Predicted correction, ΔR_pol_	Predicted correction, ΔR_pol_

**Table 4:** Distribution of training, validation, and testing samples across the five atmospheric scenes.

Atmospheric Scene	Training Samples (IDW-LUT Nodes; 70%)	Validation Samples (15%)	Test Samples (15%)	Total Samples
Clear atmosphere	28,728	6156	6156	41,040
Sulfate	28,728	6156	6156	41,040
Sea salt	28,728	6156	6156	41,040
Soot	28,728	6156	6156	41,040
Mineral dust	28,728	6156	6156	41,040
Total	143,640	30,780	30,780	205,200

**Table 5:** Hyperparameter configurations used to train the unified and scene-specific machine-learning correction models.

Method	Hyperparameter	Unified ML	Scene-Specific ML
BP	Hidden-layer widths	256, 128, 64	256, 128, 64
	Activation function	GELU	GELU
	Dropout probability	0.03	0.03
	Initial learning rate	5.0 × 10^−4^	5.0 × 10^−4^
	Weight-decay coefficient	1.0 × 10^−5^	1.0 × 10^−5^
	Mini-batch size	2048	2048
	Maximum training epochs	100	100
	Early-stopping patience (epochs)	12	12
	Gradient-norm clipping threshold	1.0	1.0
	Optimizer	AdamW	AdamW
	Loss function	MSE	MSE
HGB	Learning rate	0.04	0.04
	Number of boosting iterations	4000	4000
	Maximum leaf nodes per tree	31	31
	Maximum tree depth	No explicit limit	No explicit limit
	Minimum samples per leaf	20	20
	L2 regularization coefficient	1.0 × 10^−6^	1.0 × 10^−6^
	Maximum histogram bins	255	255
	Feature sampling fraction	1.0	1.0
	Early stopping	Disabled	Disabled
	Loss function	Squared error	Squared error
Transformer	Token embedding dimension	32	32
	Number of attention heads	4	4
	Number of encoder layers	2	2
	Feed-forward hidden dimension	128	128
	Activation function	GELU	GELU
	Dropout probability	0.05	0.05
	Initial learning rate	8.0 × 10^−4^	8.0 × 10^−4^
	Weight-decay coefficient	1.0 × 10^−5^	1.0 × 10^−5^
	Mini-batch size	2048	2048
	Maximum training epochs	50	50
	Early-stopping patience (epochs)	6	6
	Gradient-norm clipping threshold	1.0	1.0
	Optimizer	AdamW	AdamW
	Loss function	MSE	MSE

**Table 6:** Overall test-set accuracy of the scalar baseline, IDW–LUT, and unified and scene-specific machine-learning correction methods, calculated using the combined test samples from all five atmospheric scenes.

Method	Training Strategy	MARE (%)	Bias (%)	RRMSE (%)	P95 (%)	RRMSE Reduction Relative to Scalar (%)
Scalar	Baseline	2.1916	−0.0820	3.0620	5.2402	0.0000
IDW–LUT	Five scene-specific LUTs	0.5248	−0.1717	1.6872	1.4393	44.8984
BP	Unified	0.1840	−0.0102	0.6282	0.4569	79.4848
	Scene-specific	0.2693	−0.0559	0.7329	0.7301	76.0664
HGB	Unified	0.2935	−0.0078	0.5430	1.5756	82.2676
	Scene-specific	0.2978	−0.0196	0.7764	1.6966	74.6436
Transformer	Unified	0.1133	−0.0564	0.2402	0.3008	92.1547
	Scene-specific	0.2025	−0.0113	0.3050	0.6086	90.0394

Note: Let $R_{v,i}$ denote the vector-RT reference reflectance and ${\hat{R}}_{v,i}$ the corresponding scalar or corrected reflectance for test sample i. The relative error is defined as $e_{i}\;=\;100\;\frac{{\hat{R}}_{v,i}\;-\;R_{v,i}}{R_{v,i}}.$ The mean absolute relative error (MARE), bias, and relative root-mean-square error (RRMSE) are calculated as $\mathrm{MARE}\;=\;\frac{1}{N}\sum_{i=1}^{N}\left|e_{i}\right|,\;\mathrm{Bias}\;=\;\frac{1}{N}\sum_{i=1}^{N}e_{i},$ and $\mathrm{RRMSE}\;=\;\sqrt{\frac{1}{N}\sum_{i=1}^{N}e_{i}^{2}}.\;P95$ denotes the 95th percentile of $\left|e_{i}\right|$. The RRMSE reduction relative to the scalar baseline is calculated as $\mathrm{RRMSE}\;\mathrm{reduction}\;=\;100\left(1\;-\;\frac{{\mathrm{RRMSE}}_{\mathrm{method}}}{{\mathrm{RRMSE}}_{\mathrm{scalar}}}\right).$ For the correction methods, the corrected vector reflectance is reconstructed as ${\hat{R}}_{v}\;=\;R_{s}\;+\;{\hat{\Delta R}}_{\mathrm{pol}},$ whereas the scalar baseline uses $R_{s}$ directly.

**Table 7:** Testing set accuracy of the machine-learning correction models in each atmospheric scene. Accuracy was calculated separately for each scene to compare the unified and scene-specific training strategies.

Atmospheric Scene	ML Method	Training Strategy	MARE (%)	Bias (%)	RRMSE (%)	P95 (%)	RRMSE Reduction vs. Scalar (%)
Clear atmosphere	BP	Unified	0.1528	−0.0003	0.2353	0.4180	91.4198
		Scene-specific	0.1780	−0.0360	0.2991	0.4741	89.0939
	HGB	Unified	0.3002	0.0020	0.5562	1.6304	79.7163
		Scene-specific	0.2731	0.0114	0.5535	1.7757	79.8134
	Transformer	Unified	0.1067	−0.0509	0.1479	0.3005	94.6052
		Scene-specific	0.1977	0.0599	0.3070	0.6794	88.8031
Sulfate	BP	Unified	0.1470	−0.0030	0.2290	0.3995	91.6655
		Scene-specific	0.2172	−0.0620	0.3401	0.5868	87.6221
	HGB	Unified	0.2995	0.0015	0.5559	1.6316	79.7657
		Scene-specific	0.2784	0.0144	0.5565	1.7699	79.7442
	Transformer	Unified	0.0983	−0.0415	0.1372	0.2708	95.0054
		Scene-specific	0.2021	−0.1104	0.2680	0.5753	90.2469
Sea salt	BP	Unified	0.3400	−0.0674	1.3349	0.7215	67.8171
		Scene-specific	0.5146	−0.0927	1.4996	1.3908	63.8449
	HGB	Unified	0.2833	−0.0369	0.5267	1.4483	87.3018
		Scene-specific	0.3776	−0.1514	1.3389	1.5238	67.7195
	Transformer	Unified	0.1578	−0.0927	0.4557	0.3907	89.0136
		Scene-specific	0.2420	−0.1169	0.4009	0.7320	90.3354
Soot	BP	Unified	0.1367	0.0048	0.2048	0.4055	92.5316
		Scene-specific	0.2211	−0.0726	0.3621	0.5917	86.7940
	HGB	Unified	0.3002	0.0021	0.5562	1.6308	79.7168
		Scene-specific	0.2805	0.0105	0.5524	1.7384	79.8560
	Transformer	Unified	0.1040	−0.0479	0.1457	0.2940	94.6878
		Scene-specific	0.2033	0.0978	0.2905	0.5997	89.4075
Mineral dust	BP	Unified	0.1437	0.0148	0.2036	0.4125	92.3527
		Scene-specific	0.2156	−0.0162	0.3167	0.6548	88.1057
	HGB	Unified	0.2843	−0.0077	0.5186	1.5208	80.5236
		Scene-specific	0.2792	0.0171	0.5479	1.6380	79.4215
	Transformer	Unified	0.0996	−0.0491	0.1377	0.2945	94.8291
		Scene-specific	0.1676	0.0133	0.2323	0.4700	91.2737

**Table 8:** Comparison of offline preparation time, per-spectrum online computation time, and stored artifact size among DISORT, VLIDORT, IDW-LUT, and unified and scene-specific machine-learning correction methods.

Method	Total Offline Training/ Build Time (s)	Online Time (ms Per Spectrum)	Total Stored Artifact Size (MiB)
DISORT (scalar RT)	N/A	≈5.3 × 10^4^	0.2041
VLIDORT (vector RT)	N/A	≈8.7 × 10^4^	0.2051
IDW-LUT (5 scene-specific LUTs, total)	2.54	0.9892	1.3900
BP, unified (1 model)	218.45	0.2115	0.1745
BP, scene-specific (5 models, total)	494.87	0.1987	0.8480
HGB, unified (1 model)	30.02	1.3045	5.4852
HGB, scene-specific (5 models, total)	142.44	2.3960	25.7011
Transformer, unified (1 model)	1442.75	0.4636	0.1159
Transformer, scene-specific (5 models, total)	807.26	0.4429	0.5734

## Data Availability

Data underlying the results presented in this paper are not publicly available at this time but may be obtained from the authors upon reasonable request.

## Supplementary Materials

The following supporting information can be downloaded at: https://www.mdpi.com/article/10.3390/s26217084/s1, Figure S1: Wavelength-dependent polarization corrections and signed relative reflectance errors for a selected clear-atmosphere test case (NO^2^ VCD = 1 × 10^15^ molecules cm^−2^, SZA = 0°, VZA = 60°, RAA = 0°, and surface albedo = 0.40). Panels (a–c) compare the VLIDORT reference polarization correction with unified (U) and scene-specific (S) predictions from BP, HGB, and Transformer, respectively. Panels (d–f) show the corresponding signed relative reflectance errors. All 19 original wavelength samples are retained without smoothing; Figure S2: Wavelength-dependent polarization corrections and signed relative reflectance errors for a sea-salt diagnostic case. This atmospheric state was selected by searching all complete sea-salt test spectra, six machine-learning configurations, and interior wavelength points for the maximum absolute local curvature of signed relative reflectance residuals using wavelength-aware second-order finite differences. The maximum occurred for HGB-U at 436 nm. The selected state has NO^2^ VCD = 20 × 10^15^ molecules cm^−2^, SZA = 75°, VZA = 75°, RAA = 120°, and surface albedo = 0.40. Panels (a–c) compare the VLIDORT reference polarization correction with unified (U) and scene-specific (S) predictions from BP, HGB, and Transformer, respectively; panels (d–f) show signed relative reflectance errors. All 19 original wavelength samples are retained without smoothing. This deliberately selected case illustrates an extreme local spectral fluctuation, not average model performance; Supplementary Note S1: Missing-input sensitivity experiments; Figure S3: Missing-input sensitivity of the frozen unified Transformer under training-mean imputation. Panels show (a) relative root-mean-square error (RRMSE) and (b) the 95th percentile of absolute relative error (P95) over the independent test set. Nominal missingness probabilities of 0%, 5%, 10%, 20%, and 30% were applied to NO^2^ VCD, SZA, VZA, RAA, and surface albedo only; wavelength, scalar reflectance, and aerosol identity remained available. Curves show means and shaded regions indicate ±1 sample standard deviation across 30 random masks. Masks were shared across wavelengths and atmospheric scenes for each common state. Missingness refers to predictor values rather than removed samples; Table S1: Overall test accuracy under random missingness among the five eligible numerical predictors with training-mean imputation. Values are mean ± sample standard deviation over 30 random masks. All original test samples were included in every realization; standard deviations describe variability across masks, not confidence intervals; Figure S4: Test-set RRMSE of the frozen unified Transformer when one numerical predictor was unavailable for all test samples and replaced with its training-set mean. Each nonbaseline column corresponds to a single unavailable predictor, with all other inputs retained; “Complete” denotes full-input predictions. Values are reported overall and for each atmospheric scene. The heat-map color scale is logarithmic, whereas the cell labels show RRMSE (%) on the original numerical scale; Table S2: Overall test accuracy under complete inputs and single-predictor unavailability with training-mean imputation. For each unavailable-predictor condition, the indicated input was replaced for every test sample while all remaining inputs were retained. ΔRRMSE is the difference from the complete-input baseline, expressed in percentage points (pp).

## Abbreviations

The following abbreviations are used in this manuscript:

NO_2_	Nitrogen dioxide
RTM	Radiative-transfer model
LUT	Look-up table
IDW-LUT	Inverse-distance-weighted look-up table
ML	Machine learning
BP	Backpropagation neural network
HGB	Histogram-based gradient boosting
SZA	Solar zenith angle
VZA	Viewing zenith angle
RAA	Relative azimuth angle
VCD	Vertical column density
DISORT	Discrete Ordinate Radiative Transfer
VLIDORT	Vector Linearized Discrete Ordinate Radiative Transfer
MARE	Mean absolute relative error
RRMSE	Relative root-mean-square error
P95	95th percentile of absolute relative error
SSA	Single-scattering albedo
OPAC	Optical Properties of Aerosols and Clouds
TOA	Top of atmosphere
Stokes	Stokes radiance vector
