# Supplementary material for: Scene-Aware Correction of Scalar-to-Vector Reflectance in the Visible NO2 Retrieval Window
Source: Sensors (Basel). 2026 Nov 9;26(21):7084. doi: 10.3390/s26217084 (PMC13766543; doi:10.3390/s26217084)
Supplement: Supplementary file 1 [file sensors-26-07084-s001.zip › sensors-4621245-supplementary.pdf]

Scene-Aware Correction of Scalar-to-Vector Reflectance in the Visible NO_2 Retrieval Window

Supplementary Materials

Yuqian Zhang[1,2], **Kun Wu**[1,*] **and Yijing Lu**[3]

1 Collaborative Innovation Center on Forecast and Evaluation of Meteorological Disasters (CIC-FEMD) / State Key Laboratory of Climate System Prediction and Risk Management (CPRM) / Earth System Modeling Center, Nanjing University of Information Science and Technology, Nanjing 210044, China

2 Department of Earth and Planetary Sciences, Institute for Atmospheric and Climate Science, ETH Zürich, Zürich 8092, Switzerland

3 Department of Earth and Atmospheric Sciences, Cornell University, Ithaca 14853, United States

* Correspondence: wukun@nuist.edu.cn

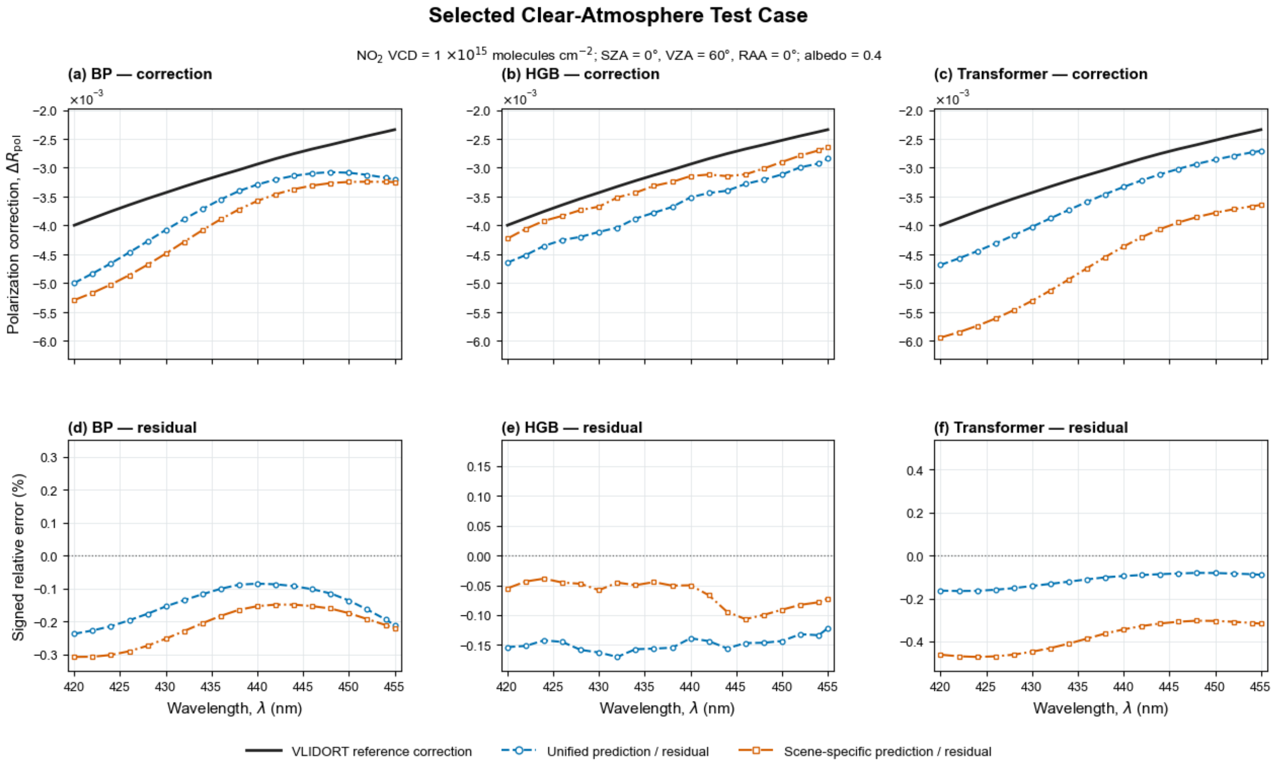

Figure S1. Wavelength-dependent polarization corrections and signed relative reflectance errors for a selected clear-atmosphere test case (NO_2 VCD = 1 × 10^{15} molecules cm^{-2}, SZA = 0°, VZA = 60°, RAA = 0°, and surface albedo = 0.40). Panels (a–c) compare the VLIDORT reference polarization correction with unified (U) and scene-specific (S) predictions from BP, HGB, and Transformer, respectively. Panels (d–f) show the corresponding signed relative reflectance errors. All 19 original wavelength samples are retained without smoothing.

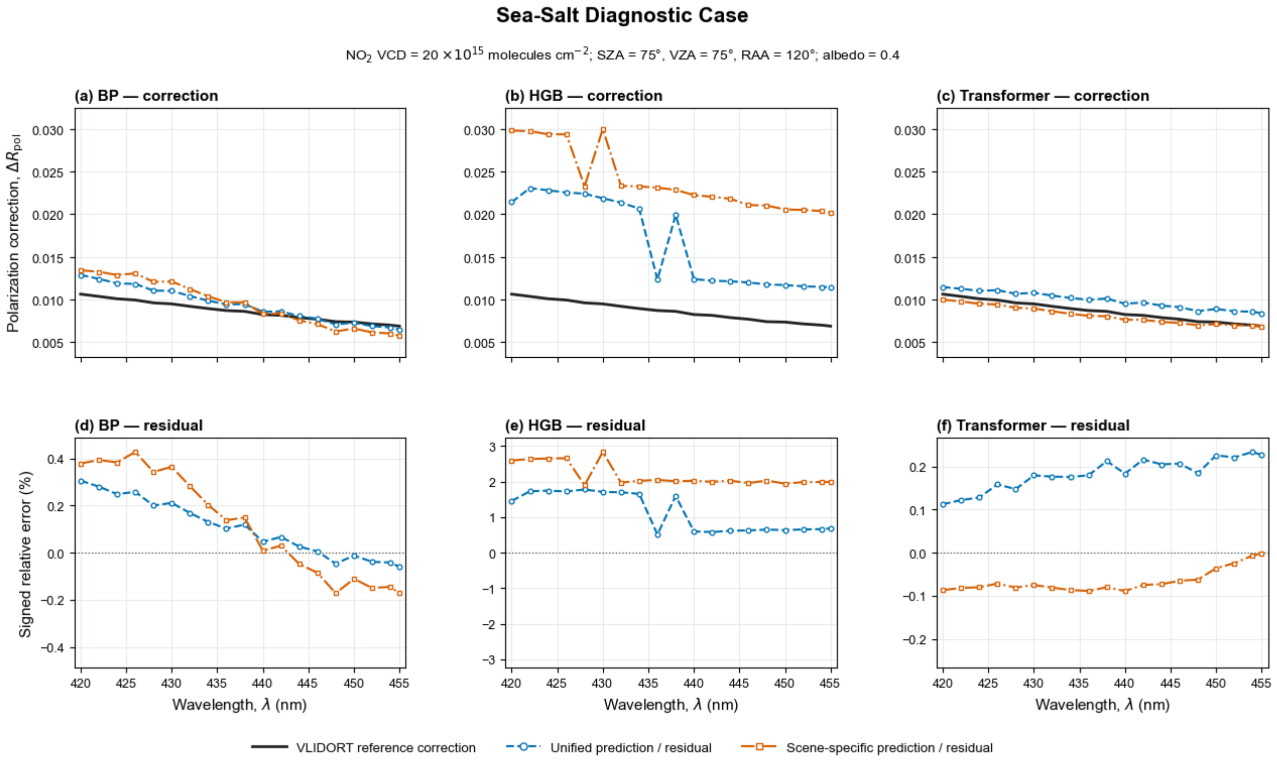

Figure S2. Wavelength-dependent polarization corrections and signed relative reflectance errors for a sea-salt diagnostic case. This atmospheric state was selected by searching all complete sea-salt test spectra, six machine-learning configurations, and interior wavelength points for the maximum absolute local curvature of signed relative reflectance residuals using wavelength-aware second-order finite differences. The maximum occurred for HGB-U at 436 nm. The selected state has NO_2 VCD = 20 × 10^{15} molecules cm^{-2}, SZA = 75°, VZA = 75°, RAA = 120°, and surface albedo = 0.40. Panels (a–c) compare the VLIDORT reference polarization correction with unified (U) and scene-specific (S) predictions from BP, HGB, and Transformer, respectively; panels (d–f) show signed relative reflectance errors. All 19 original wavelength samples are retained without smoothing. This deliberately selected case illustrates an extreme local spectral fluctuation, not average model performance.

Supplementary Note S1. Missing-input sensitivity experiments

Evaluation framework. The original unified Transformer (Transformer-U) was held fixed and evaluated using the same independent test partition as in the main manuscript. No model parameters, hyperparameters, or radiative-transfer simulations were changed. Inference was performed in evaluation mode, with dropout disabled. Predictions with complete inputs reproduced the archived model performance (RRMSE = 0.2402%, MARE = 0.1133%, and P95 = 0.3008%).

Eligible predictors and imputation. Synthetic missingness was introduced only for five numerical predictors: NO_2 vertical column density (VCD), solar zenith angle (SZA), viewing zenith angle (VZA), relative azimuth angle (RAA), and surface albedo. Wavelength, scalar reflectance (R_s), and aerosol identity remained available. The reference vector reflectance (R_v) was used only for performance evaluation. Each missing numerical value was replaced with the corresponding training-set mean stored with the original model; the original standardization was then applied. The training means were 9.0000×10^{15} molecules cm^{-2} (NO_2 VCD), 37.5000° (SZA), 37.5000° (VZA), 90.2381° (RAA), and 0.1900 (surface albedo). After standardization, an imputed value becomes zero, but the original physical quantity is not set to zero. No missingness indicator or additional input token was provided to the Transformer.

Random-missingness protocol. Each of the five eligible predictors was masked with nominal probabilities of 0%, 5%, 10%, 20%, and 30%. Independent masks were generated for each shared NO_2-VCD–geometry–surface state and predictor, then applied consistently to all corresponding wavelengths and atmospheric scenes. Thirty independent random-mask realizations were evaluated at each probability. Missingness represents unavailable predictor values, not removal of test samples; the entire independent test set remained in every realization. The frozen model was evaluated on the imputed inputs without retraining.

Single-predictor protocol. In a separate stress test, one of the five eligible predictors was unavailable for every test sample and replaced by its training-set mean, while the remaining inputs were retained. Five such conditions were compared with the complete-input baseline. This protocol measures the effect of losing an entire predictor, and is distinct from random missingness at the same nominal proportion.

Accuracy summaries. The signed relative reflectance error and the RRMSE, MARE, and P95 metrics follow the definitions in the main manuscript. For random missingness, the performance metrics were calculated separately for each random-mask realization and then summarized as the mean ± sample standard deviation across 30 realizations. The shaded regions in Figure S3 represent ±1 standard deviation across masks, not confidence intervals. The single-predictor results are deterministic for the chosen inputs and imputation values.

Scope. These experiments assess the existing Transformer-U combined with training-mean imputation; they do not establish native missing-value handling, and they do not evaluate missing wavelength, missing scalar reflectance, missing aerosol identity, or alternative missingness mechanisms. In particular, changes under NO_2-VCD imputation should not be interpreted as evidence that NO_2 absorption is physically unimportant.

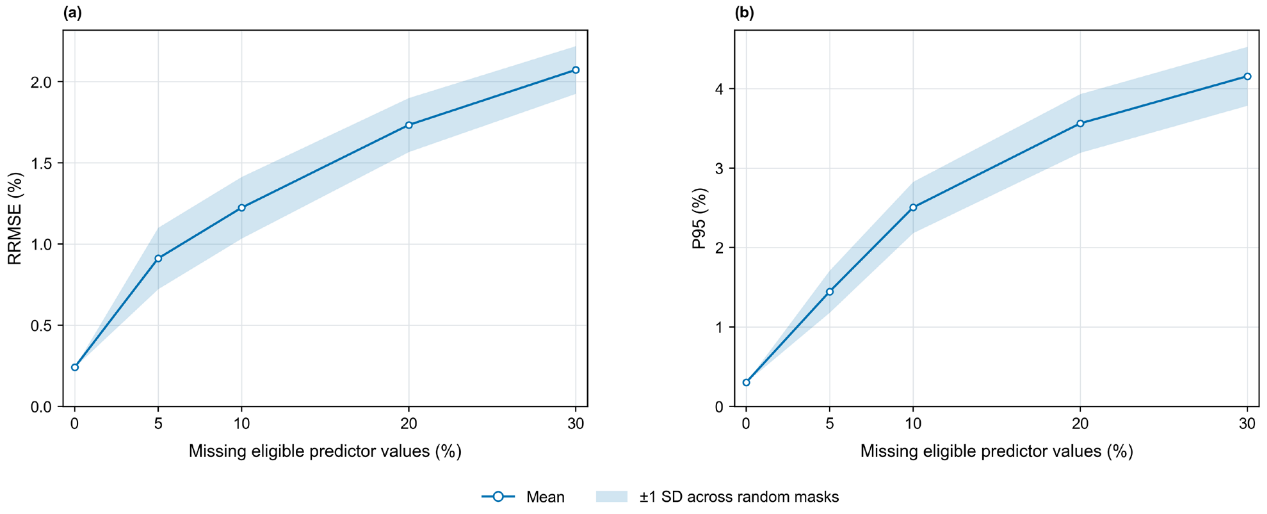

Figure S3. Missing-input sensitivity of the frozen unified Transformer under training-mean imputation. Panels show (a) relative root-mean-square error (RRMSE) and (b) the 95th percentile of absolute relative error (P95) over the independent test set. Nominal missingness probabilities of 0%, 5%, 10%, 20%, and 30% were applied to NO_2 VCD, SZA, VZA, RAA, and surface albedo only; wavelength, scalar reflectance, and aerosol identity remained available. Curves show means and shaded regions indicate ±1 sample standard deviation across 30 random masks. Masks were shared across wavelengths and atmospheric scenes for each common state. Missingness refers to predictor values rather than removed samples.

Table S1. Overall test accuracy under random missingness among the five eligible numerical predictors with training-mean imputation. Values are mean ± sample standard deviation over 30 random masks. All original test samples were included in every realization; standard deviations describe variability across masks, not confidence intervals.

Nominal missingness	RRMSE (%)	MARE (%)	P95 (%)
0%	0.2402 ± 0.0000	0.1133 ± 0.0000	0.3008 ± 0.0000
5%	0.9116 ± 0.1892	0.3021 ± 0.0341	1.4456 ± 0.2647
10%	1.2242 ± 0.1896	0.4783 ± 0.0490	2.5054 ± 0.3224
20%	1.7337 ± 0.1664	0.8206 ± 0.0552	3.5630 ± 0.3695
30%	2.0731 ± 0.1467	1.1101 ± 0.0554	4.1570 ± 0.3705

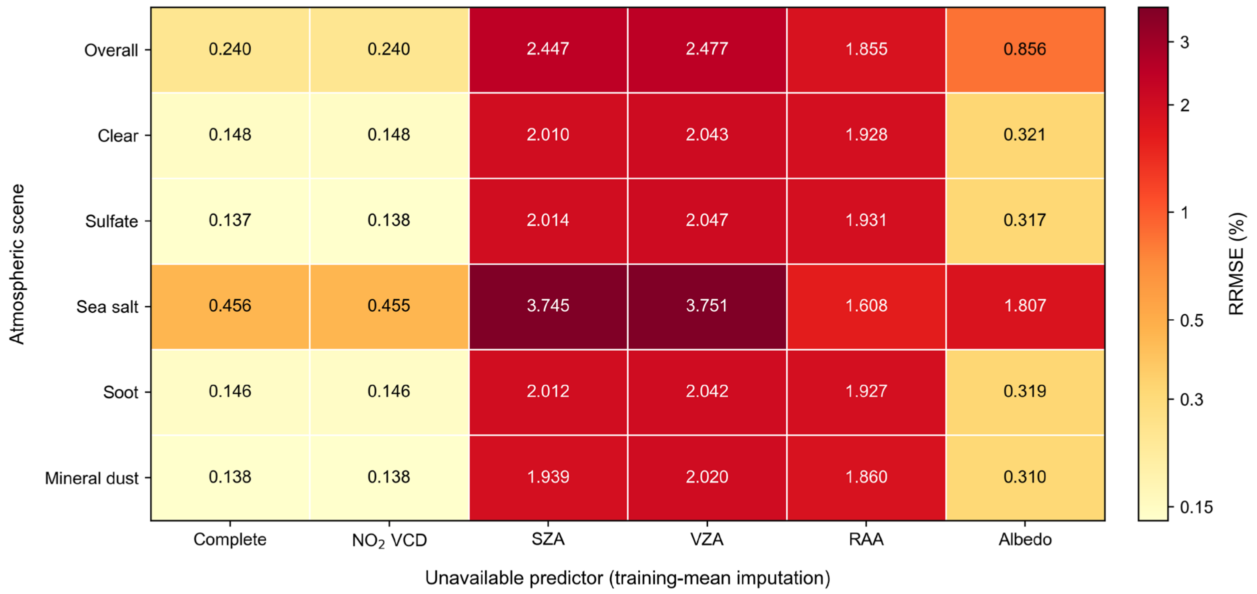

Figure S4. Test-set RRMSE of the frozen unified Transformer when one numerical predictor was unavailable for all test samples and replaced with its training-set mean. Each nonbaseline column corresponds to a single unavailable predictor, with all other inputs retained; “Complete” denotes full-input predictions. Values are reported overall and for each atmospheric scene. The heat-map color scale is logarithmic, whereas the cell labels show RRMSE (%) on the original numerical scale.

Table S2. Overall test accuracy under complete inputs and single-predictor unavailability with training-mean imputation. For each unavailable-predictor condition, the indicated input was replaced for every test sample while all remaining inputs were retained. ΔRRMSE is the difference from the complete-input baseline, expressed in percentage points (pp).

**Input condition**	**RRMSE (%)**	**MARE (%)**	**P95 (%)**	**ΔRRMSE (pp)**
Complete inputs	0.2402	0.1133	0.3008	+0.0000
NO_2 VCD unavailable	0.2401	0.1137	0.3041	−0.0001
SZA unavailable	2.4467	1.4574	4.5216	+2.2065
VZA unavailable	2.4773	1.5689	4.2865	+2.2370
RAA unavailable	1.8550	1.1459	4.8003	+1.6148
Albedo unavailable	0.8563	0.2533	0.7985	+0.6161
